# The determination of human creative thinking by employing machine learning classification on EEG signals

**DOI:** 10.3389/fpsyg.2026.1809948

**Published:** 2026-07-02

**Authors:** Jilin Zou, Fang Yuan, Silin Zhou, Jiaqin Yang, Chunlei Liu

**Affiliations:** 1 Department of Psychology, Linyi University, Linyi, Shandong, China; 2 School of Psychology, Qufu Normal University, Qufu, Shandong, China

**Keywords:** creative thinking, EEG, entropy, machine learning, power spectral density, support vector machine

## Abstract

**Background and objective:**

Traditional creativity assessments are limited by subjectivity and high labor costs. Although machine learning (ML) offers objective alternatives, its application to EEG-based creativity evaluation remains scarce. This study aimed to classify high and low creative thinking from EEG signals using ML.

**Methods:**

One hundred forty participants completed the Alternative Uses Task during EEG recording. Three independent raters (none were authors) evaluated response originality using the Consensus Assessment Technique on a 1-to-5 scale; mean scores were dichotomized at the median into high- and low-creativity labels (996 and 1,096 trials, respectively, from 2,092 valid trials). Classification features included alpha-band Power Spectral Density (PSD), Approximate Entropy, Sample Entropy, and a combined feature set. Six classifiers—Support Vector Machine (SVM), Quadratic Discriminant Analysis (QDA), Logistic Regression (LogR), Decision Tree (DT), XGBoost, and LightGBM—were trained and evaluated using a 10-fold cross-validation strategy. To prevent subject-level information leakage, a Leave-One-Subject-Out (LOSO) validation was additionally conducted.

**Results:**

All six classifiers effectively distinguished creativity levels. Under 10-fold cross-validation, SVM achieved optimal performance using Approximate Entropy or Sample Entropy (*F*1-score = 90.5%; accuracy = 89.8%). The combined feature set yielded comparable results. LOSO validation confirmed generalizability to unseen individuals, with SVM attaining *F*1-scores of 82.4% (Approximate Entropy) and 82.1% (Sample Entropy). Entropy-based features consistently outperformed alpha PSD.

**Conclusion:**

ML effectively classifies creativity from EEG signals. The superior performance of entropy features, supported by both trial-level and subject-independent validation, highlights the robustness of the proposed approach and its potential for developing objective, scalable creativity assessment tools.

## 1 Introduction

Creativity, a higher cognitive function of the brain, is defined as an individual’s ability to generate novel and appropriate ideas or products within a specific context (Runco and Jaeger, 2012). The specific manifestation of creativity is known as creative thinking. To date, there is no single, precise definition of creative thinking due to the wide variation in its interpretation and application among different psychologists. Notably, most researchers agree on the characteristics of uniqueness, novelty, and flexibility that define creative thinking (Liu et al., 2009; Zhuang, 2001). Creative assessment plays a crucial role in the research of creative thinking.

Currently, creativity is usually assessed using divergent thinking tasks, such as the Alternative Uses Task (AUT). This task requires participants to generate as many novel uses as possible for common items, such as “bricks” (Gilhooly et al., 2007), within a specified time limit. It has demonstrated that the AUT effectively differentiates between individuals with high and low creativity (Benedek, 2018; Orwig et al., 2021). However, traditional creativity assessments necessitate that a rater manually score the responses. Even when evaluating the same item, raters do not always agree on what constitutes a creative use. To mitigate this subjectivity, the common approach is to increase the number of raters and estimate creativity by averaging the ratings. However, this method also introduces the challenge of significant labor costs (Yang et al., 2023).

Machine Learning (ML) methods can effectively address the limitations of traditional creativity assessments. ML is the study of computer algorithms that enable computers to automatically learn from data and prior experiences, allowing them to discover patterns and make predictions without human intervention (Mahesh, 2020; Soori et al., 2023). ML provides innovative tools for neuroscience research (Hosseini et al., 2021). The ability to incorporate the regular information inherent in data-rich EEG signals enhances the capacity to decode brain functions and uncover complex neural data patterns (Huang et al., 2023). Currently, the applications of ML in EEG signal processing have expanded to several fields, including mental illness classification, emotion recognition, and sleep disorder diagnosis (Bracher-Smith et al., 2022; Horigome et al., 2020; Kumar et al., 2022; Nassehi et al., 2024; Salama et al., 2018). These applications highlight the versatility of machine learning techniques in extracting meaningful information from EEG signals. While the integration of EEG signals with ML techniques has achieved considerable success in cognitive science, its application in creative thinking research remains relatively scarce (Sasaki et al., 2019; Stevens and Zabelina, 2020). Moreover, most studies have used raw EEG data as model inputs, which typically exhibit high signal-to-noise ratios. Consequently, there is a pressing need to further analyze EEG data to investigate the impact of new classification features on the accuracy of creative classification.

**Figure 1:**
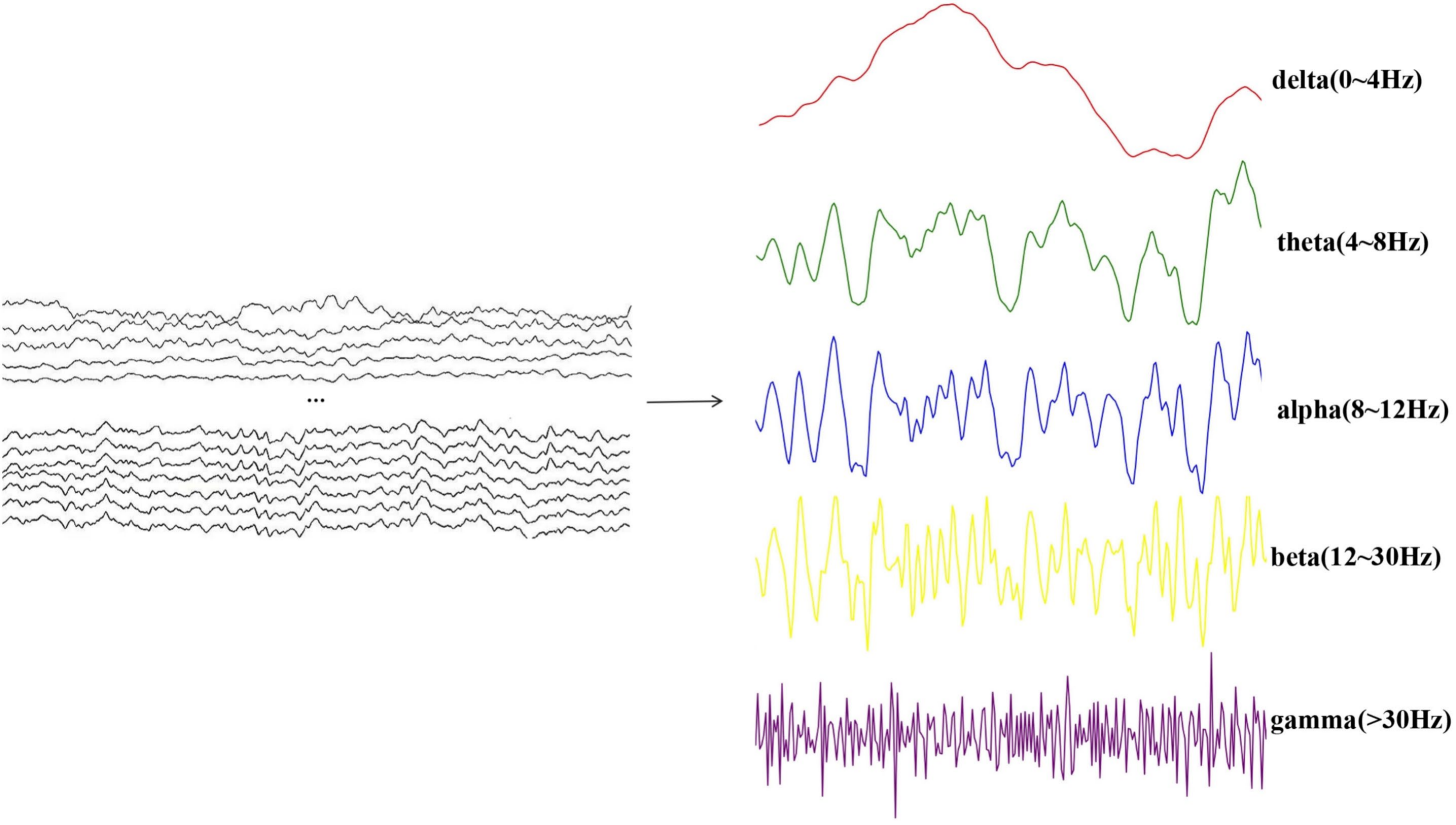
EEG bands.

Among the EEG bands (see Figure 1), the correlation between the alpha band and creative thinking has been consistently supported by research (Fink et al., 2009; Fink and Benedek, 2014). Individuals with high levels of creativity exhibit greater alpha band energy compared to those with lower levels of creativity (Fink and Neubauer, 2008). In addition to the traditional time-frequency domain, researchers have recently incorporated nonlinear analysis into EEG studies, such as information entropy. This concept, first introduced by Shannon, quantifies the amount of information in a dataset, with higher information entropy indicating greater information content. To accommodate various types of data and domain requirements, as well as to further characterize the variation in information content, Pincus proposed the dynamic parameter known as “approximate entropy” to measure the complexity of signals from a time series perspective. Building on this foundation, Richman and Moorman (2000) developed a more accurate complexity measurement method called “sample entropy”. Sample entropy offers several advantages over approximate entropy, including reduced computational errors, independence from data length, and enhanced consistency. Both sample entropy and approximate entropy are robust against noise, effectively capture the complexity of EEG signals, and are associated with creativity across various EEG analyses (Krishnan et al., 2020; Ueno et al., 2015).

**Figure 2:**
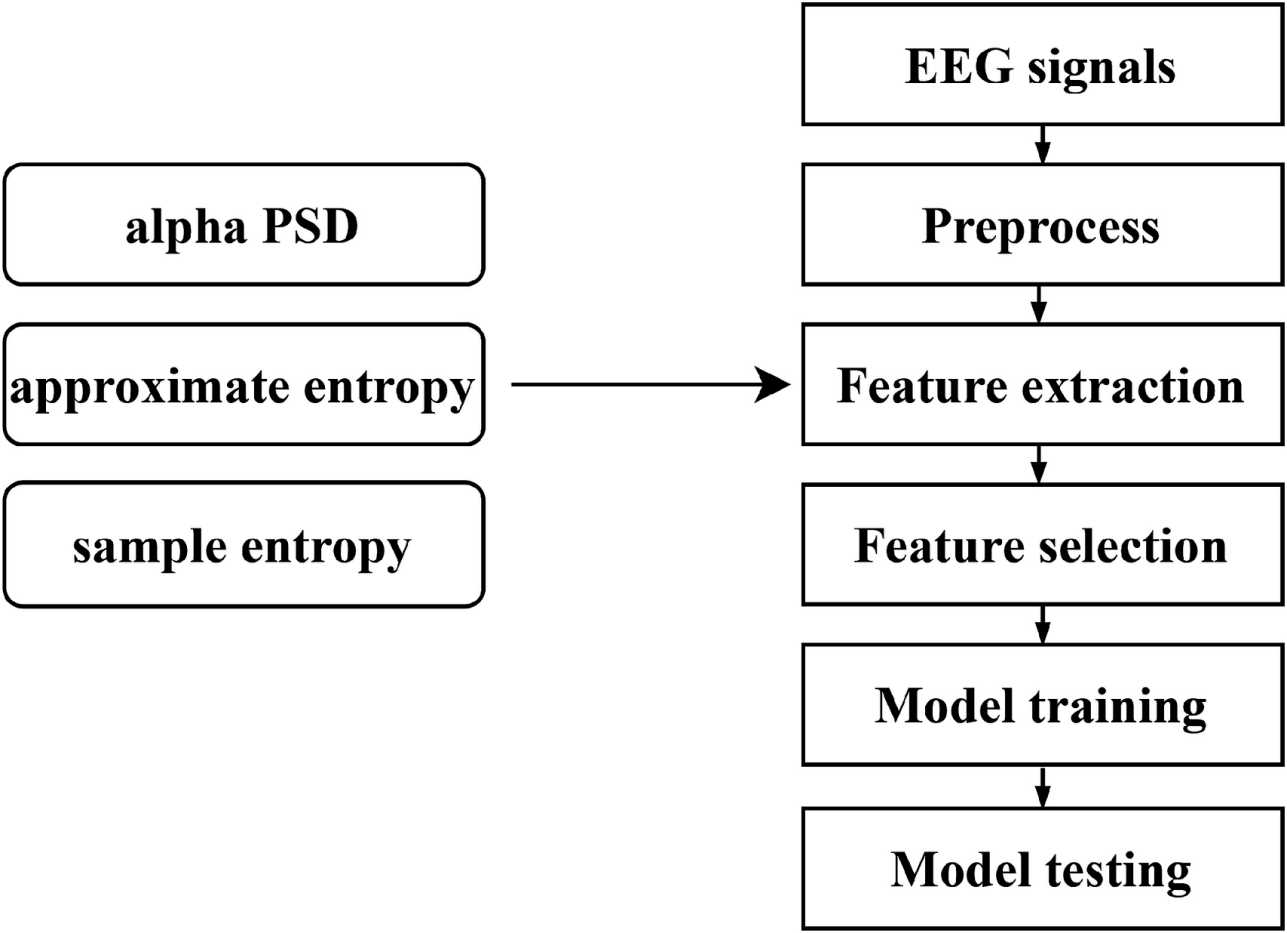
Machine learning flowchart.

Therefore, in this study, we selected the power spectral density (PSD) of the alpha band (Thilakavathi et al., 2019), approximate entropy, and sample entropy (Delgado-Bonal and Marshak, 2019) as classification features (see Figure 2), while the scoring results of the AUT were used as labels (high and low). To facilitate a better comparison of the classification performance among different machine learning models applied to creative thinking EEG data, we explored the optimal feature-algorithm combinations for creative classification using support vector machines (SVM), quadratic discriminant analysis (QDA), logistic regression (LogR), decision trees (DT), extreme gradient boosting (XGBoost), and Light Gradient Boosting Machine (LightGBM).

## 2 Method

### 2.1 Participants

In G*Power 3.1.9.7 software (Faul et al., 2009), a medium effect size (*d* = 0.5) and a significance level (*α* = 0.05) were established, with an expected statistical power of 80%. This indicated that a minimum of 126 participants was required. A total of 141 college students were recruited for the study. However, one participant was excluded due to an obviously irrelevant response. The mean age of the remaining participants (*n* = 140; 71 females) was 20.65 ± 1.86 years (mean ± standard deviation). All participants were right-handed, native Chinese speakers, and had normal vision or corrected-to-normal vision. The study received approval from the Ethics Committee of Qufu Normal University, and all participants signed an informed consent form prior to the experiment. Upon completion of the experiment, participants were compensated in accordance with the established protocol.

### 2.2 Procedure

**Figure 3:**
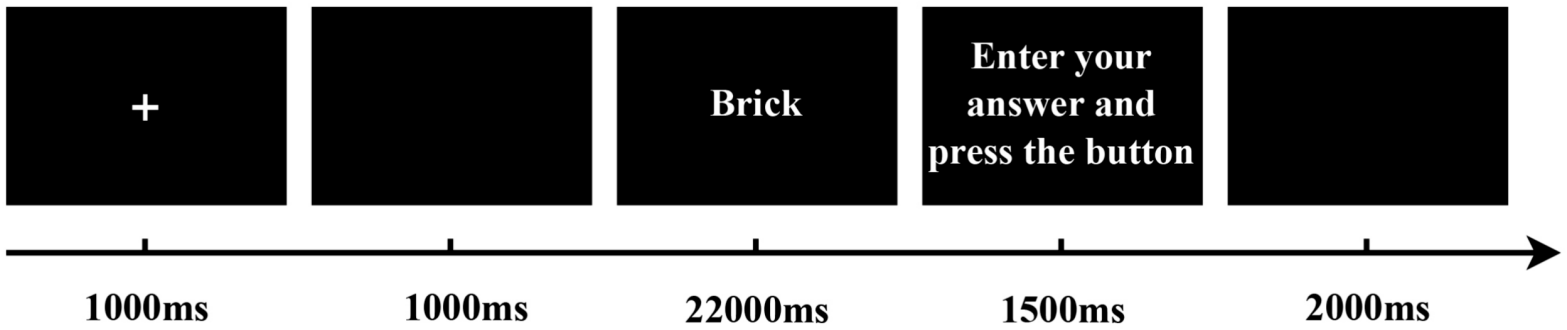
Trial structure of the alternate uses task (AUT).

The stimuli were presented using E-Prime 2.0. Participants completed the Chinese version of the AUT during EEG recording (see Figure 3). They were instructed to generate as many creative applications for the given object as possible within a specified time frame and then select the most original and appropriate idea. The experimental procedure began with the presentation of a fixation cross (“+”) on the screen for 1,000 ms, followed by a blank screen for an additional 1,000 ms. Subsequently, participants were shown the name of an everyday object for 22,000 ms and asked to generate as many creative uses for that object as they could. After this stimulation period, participants were prompted to provide the most creative use they could think of, with a response interval of 2,000 ms. Each participant was required to complete 15 trials, divided into three blocks, with a self-determined rest period between each block. Prior to the commencement of the formal experiment, participants completed practice trials to familiarize themselves with the task rules and procedures.

For creativity scoring, we employed the consensual assessment technique (Amabile, 1982). Three independent and experienced raters evaluated each participant’s creative response for each everyday object on a 5-point scale (1 = not creative at all; 5 = highly creative). None of the raters are authors of this manuscript. All participant responses were transcribed into a spreadsheet, assigned unique identifiers known only to the experimenters, and then randomized to ensure that raters were blind to response sequence, total number, and participant identity. Prior to formal scoring, raters completed a brief practice session by independently evaluating a set of sample responses (not included in the dataset) on the 5-point scale to familiarize themselves with the procedure. During the formal evaluation, the three independent raters assessed the originality of each response (e.g., Agnoli et al., 2020). Scores from the three raters were averaged to yield an originality score for each trial. The inter-rater reliability (ICC) of the creativity ratings was satisfactory (ICC = 0.863, 95% CI [0.779, 0.908]). Finally, participants’ originality scores were averaged across all items to obtain their final creativity score.

The full distribution of originality scores across all 2,092 trials is presented in Table 1. The median originality score was 2.644 which was used as the threshold for classification: trials with scores ≥ median were classified as high creativity (*n* = 996), and trials with scores <median were classified as low creativity (*n* = 1,096). Eight trials were missing due to an interruption in data recording, leading to a final total of 2,092 trials.

**Table 1:** Distribution of originality scores across all trials (*N* = 2,092).

Originality score	1 (lowest)	2	3	4	5 (highest)
Number of trials	113	858	860	183	78
Percentage	5.4%	41.01%	41.11%	8.75%	3.73%

### 2.3 EEG recording and preprocessing

EEG signals were acquired using a Brain Products ActiCHamp active channel amplifier and actiCAP 64 conductive electrode caps arranged according to the international 10–20 system. The sampling rate was set to 1,000 Hz, and the online filters were configured with a bandwidth of 0.1 to 100 Hz, including notch filters at 50 Hz. The FCz electrode was selected as the reference, while the FPz electrode served as the ground. It was ensured that the impedance of all electrodes remained below 5 KΩ. The EEG signal data were preprocessed using EEGLAB version 14.2.1 (Delorme and Makeig, 2004), executed within MATLAB 2021a (MathWorks, United States). Binaural mastoid averaging was employed for re-referencing, with band-pass filtering applied between 0.1 and 40 Hz. Artifacts, such as eye movements and electromyographic (EMG) signals, were corrected through independent component analysis (ICA; (Delorme and Makeig, 2004)). Artifacts exhibiting amplitude fluctuations exceeding ±50 μV between sampling points, amplitude differences greater than ±200 μV between peaks occurring within a latency of 200 ms, amplitude differences exceeding ±100 μV between maximum and minimum amplitudes, and amplitude fluctuations less than ±0.5 μV over a period of 100 ms were removed.

### 2.4 EEG features

EEG features were calculated using MATLAB 2021a software.

#### 2.4.1 Power spectral density

The EEG sequence *x* [*n*] is windowed by window sequence *w* [*n*] with *R* overlapping portions of length *M*. Let the overlap between adjacent samples be kept as *K* samples. Then the windowed *r*th segment of the data *x* [*n*] is represented using Equation 1:


$$x_{r}\left[n\right]=x\left[n+rK\right],0\leq n\leq M-1,0\leq r\leq R-1$$ (1)

EEG waves are non-stationary, it is essential to consider piecewise stationary portion of the signal for analysis. So, 2.56 s epochs are considered as a stationary wave. Windowing technique is done using Equation 2 with hamming window *w* (*n*) of length 2092 samples (2.56 s) to control the abruptness of the transition to zero with 50% (1,046 samples) overlap.


$$x_{r}\left[N\right]=w\left(n\right)x\left[n+rK\right]$$ (2)

The shape of the hamming window is almost always decreasing to zero at boundaries. Hence there is a possibility of losing some information. In order to avoid information loss, overlapping of windows are allowed with 50% overlapping. For each $x_{r}\left[N\right]$ , the power spectrum is calculated using Equation 3 and it is represented as $P_{r}xx\left(e^{jw}\right)$ .


$$P_{r}xx\left(e^{jw}\right)=\frac{1}{RM}\overset{R-1}{\underset{r=0}{\sum }}{\left|\overset{M-1}{\underset{n=0}{\sum }}w\left(n\right)x\left(n+rK\right)e^{-jkw}\right|}^{2}$$ (3)

$P_{r}xx\left(e^{jw}\right)$ is calculated for all *R* overlapping portions and the Welch estimate is then given by the average of all *R* periodogram using Equation 4. This is considered as a absolute power of a given spectrum.


$$Pxx\left(e^{jw}\right)=\frac{1}{R}\overset{R-1}{\underset{r=0}{\sum }}P_{r}xx\left(e^{jw}\right)$$ (4)

#### 2.4.2 Approximate entropy

Given a sequence of numbers u={u(1),u(2),…,u(N)} of length *N*, a non-negative integer *m* = 2, with *m* ≤ *N* and a positive real number *r* = 0.2, we define the blocks x(i)={u(i),u(i+1),…,u(i+m−1)} and x(j)={u(j),u(j+1),…,u(j+m−1)}, and calculate the distance between them as $d\left[x\left(i\right),x\left(j\right)\right]=\max_{k=1,2,\ldots ,m}\left(u\left(i+k-1\right)-u\left(j+k-1\right)\right)$ . Then we calculate the value $C_{i}^{m}\left(r\right)=\left(\left(\mathrm{number}\;\mathrm{of}j\leq N-m+1\mathrm{such}\;\mathrm{that}d\left[x\left(i\right),x\left(j\right)\right]\leq r\right)/\left(N-m+1\right)\right).$ The numerator of $C_{i}^{m}$ counts, within the resolution *r*, the number of blocks of consecutive values of length *m* which are similar to a given block.


$$\phi^{m}\left(r\right)=\frac{1}{N-m+1}\overset{N-m+1}{\underset{i=1}{\sum }}\log C_{i}^{m}\left(r\right)$$ (5)

We can define ApEn (*m*, *r*, *N*) (*u*) = *φ^m^* (*r*) − *φ*^*m*+1^ (*r*), with *m* ≥ 1 and ApEn (0, *r*, *N*) (*u*) = −*φ*^1^ (*r*). ApEn (*m*, *r*, *N*) (*u*) measures the logarithmic frequency with which blocks of longitude *m* that are close together stay together for the next position, or put differently, the negative value of approximate entropy is defined as:

−ApEn (*m*, *r*, *N*) (*u*) = *φ*^*m*+1^ (*r*) − *φ^m^* (*r*) = average over *i* of the logarithm (conditional probability of |*u* (*j* + *m*) – *u* (*i* + *m*)| ≤ *r*, if it is verified that |*u* (*j* + *k*) – *u* (*i* + *k*)| ≤ *r* for *k* = 0, 1, …, *m* − 1).

ApEn (*m*, *r*, *N*) is the statistical estimator of the parameter ApEn (*m*, *r*) (Equations 5 and 6):


$$\mathrm{ApEn}\left(m,r\right)=\underset{N\to \infty }{\lim}\left[\phi^{m}\left(r\right)-\phi^{m+1}\left(r\right)\right]$$ (6)

ApEn (*m*, *r*, *N*) is a family of statistics and the comparisons between systems are intended with fixed values of *m* and *r*, and, if possible, with the same number of observations *N* due to the bias that we will mention later. Approximate entropy measures the likelihood that runs of patterns that are close for m observations remain close on next incremental comparisons. Greater likelihood of remaining close, implying regularity, produces smaller approximate entropy values, and conversely (Pincus and Goldberger, 1994). Ultimately, the conditional probabilities in the correlation integral determine the value of approximate entropy.

#### 2.4.3 Sample entropy

We define the total number of possible vectors by calculating for each template vector, *m* = 2 and *r* = 0.2:


$$\begin{array}{c} B_{i}^{m}\left(r\right)=\frac{1}{N-m+1}\times \left[\begin{array}{l} \mathrm{number}\;\mathrm{of}\;\mathrm{vectors}x_{m}\left(j\right)\mathrm{at}a\\ \mathrm{distance}r\mathrm{of}x_{m}\left(i\right),\;\mathrm{without}\;\mathrm{allowing}\;\\ \mathrm{self}-\mathrm{counting,}\;\mathrm{where}j=1,N-m \end{array}\right]\\ =\frac{1}{N-m+1}\overset{N-m}{\underset{j=1,j\neq i}{\sum }}\left[\begin{array}{l} \mathrm{number}\;\mathrm{of}\;\mathrm{times}\;\mathrm{that}d\\ \left[x_{m}\left(j\right)-x_{m}\left(i\right)\right]<r \end{array}\right] \end{array}$$ (7)

and adding all the template vectors:


$$\begin{array}{l} B^{m}\left(r\right)=\frac{1}{N-m}\overset{N-m}{\underset{i=1}{\sum }}B_{i}^{m}\left(r\right)=\frac{1}{N-m-1}\frac{1}{N-m}\\ \overset{N-m}{\underset{i=1}{\sum }}\overset{N-m}{\underset{j=1,j\neq i}{\sum }}\left[\begin{array}{l} \mathrm{number}\;\mathrm{of}\;\mathrm{times}\;\mathrm{that}d\\ \left[x_{m}\left(j\right)-x_{m}\left(i\right)\right]<r \end{array}\right] \end{array}$$ (8)

In the same way, we define the total number of matches by calculating for each model vector:


$$\begin{array}{l} A_{i}^{m}\left(r\right)=\frac{1}{N-m-1}\times \left[\begin{array}{l} \mathrm{number}\;\mathrm{of}\;\mathrm{vectors}x_{m+1}\left(j\right)\mathrm{at}a\\ \mathrm{distance}r\mathrm{of}x_{m+1}\left(i\right),\;\mathrm{without}\;\\ \mathrm{allowing}\;\mathrm{self}-\mathrm{counting,}\;\\ \mathrm{where}j=1\mathrm{,,,}N-m \end{array}\right]\\ =\frac{1}{N-m-1}\overset{N-m}{\underset{j=1,j\neq i}{\sum }}\left[\begin{array}{l} \mathrm{number}\;\mathrm{of}\;\mathrm{times}\;\mathrm{that}d\\ \left[\left|x_{m+1}\left(j\right)-x_{m+1}\left(i\right)\right|\right]<r \end{array}\right] \end{array}$$ (9)

and adding them as:


$$\begin{array}{l} A^{m}\left(r\right)=\frac{1}{N-m}\overset{N-m}{\underset{i=1}{\sum }}A_{i}^{m}\left(r\right)=\frac{1}{N-m-1}\frac{1}{N-m}\\ \overset{N-m}{\underset{i=1}{\sum }}\overset{N-m}{\underset{j=1,j\neq i}{\sum }}\left[\begin{array}{l} \mathrm{number}\;\mathrm{of}\;\mathrm{times}\;\mathrm{that}d\\ \left[x_{m+1}\left(j\right)-x_{m+1}\left(i\right)\right]<r \end{array}\right] \end{array}$$ (10)

Therefore, $B^{m}\left(r\right)$ is the probability that two sequences are similar for *m* points (possibles), while $A^{m}\left(r\right)$ is the probability that two sequences are similar for m+1 points (matches). Since the number of matches is always less than or equal to the number of possible vectors, the ratio $A^{m}\left(r\right)$ / $B^{m}\left(r\right)$ is a conditional probability less than unity. (Equations 7-10).

The parameter Sample Entropy is defined as $\mathrm{SampEn}\left(m,r\right)=\lim_{N\to \infty }\left\{-\log\left[A^{m}\left(r\right)/B^{m}\left(r\right)\right]\right\}$ , value which is estimated from the statistic $\mathrm{SampEn}\left(m,r\right)=-\log\left[A^{m}\left(r\right)/B^{m}\left(r\right)\right]$ .

#### 2.4.4 Feature selection

In this study, the recursive feature elimination (RFE) algorithm, part of the wrapper feature selection method, is employed for feature selection (Yi et al., 2022). This approach aims to eliminate redundant features and identify those most effective for categorizing high and low creativity groups. Initially, the weight of each feature is calculated using the RFE algorithm, which involves randomly selecting samples and identifying adjacent samples within each category to update the feature weights. The features are then ranked from highest to lowest weight and sequentially input into the classification model. Each time the input feature subset changes, ten-fold cross-validation is performed to determine the mean classification accuracy of the current feature subset within the model. When the nth new feature is added to the *N*th feature subset, a new mean classification accuracy is obtained following model training. Subsequently, the average accuracy of the *N*th feature subset is compared with that of the (*N* − 1)th feature subset. If the average accuracy of the *N*th feature subset exceeds that of the (*N* − 1)th subset, the *n*th feature is included in the optimal feature subset; otherwise, it is discarded. The RFE algorithm continues this process, traversing all features to ultimately identify the optimal feature subset.

The subset of features for PSD included: C3, T7, P3, P4, P8, C4, FC2, F8, AF3, FT7, C1, C5, TP7, CP3, P1, P5, PO7, POz, PO4, PO8, P2, CPz, CP4, TP8, C2, FT8, F6, AF4, F2. The subset of features for approximate entropy included: P7, P4, FC6, F5, FT7, P5, PO3, PO4, F2. The subset of features for sample entropy included: FC6, F8, AF7, AFz, F1, TP7, P1, PO7, PO3, P2, CPz, C2.

### 2.5 General statistical analysis

General statistical analysis was conducted using IBM SPSS Statistics 27.0.1. Differences between the high and low creativity groups regarding the feature subsets of PSD, approximate entropy, and sample entropy were analyzed using the Welch-corrected independent samples *t*-test. ML metrics were compared using the Friedman test, followed by the Bonferroni post-hoc test (He et al., 2025). A two-sided significance level of *α* = 0.05 was employed.

### 2.6 Machine learning analysis

In this study, six classification models—SVM, QDA, LogR, DT, XGBoost, and LightGBM—are employed to evaluate their performance using a 10-fold cross-validation strategy. Specifically, 80% of the dataset is allocated to a training set, while the remaining 20% is designated as a test set. Next, 10-fold cross-validation is conducted on the training set; the training set is randomly divided into 10 subsets. In each iteration, one subset is selected as the validation set, while the other nine subsets serve as the training set. This process is repeated 10 times to train 10 distinct models. Subsequently, the average performance index of these 10 models is calculated, which serves as the foundation for model tuning, allowing for the selection of the best-performing parameters as the optimal settings.

After determining the optimal parameters, the model is trained on the training set using this configuration, and it is subsequently validated on the test set to obtain the final performance metrics. Based on this optimal parameter setting, we randomly repeated the model construction 50 times, each time allocating 80% of the data for the training set and 20% for the test set. Finally, the performance results obtained from these 50 iterations are averaged to provide the training results for this machine learning classifier model.

**Figure 4:**
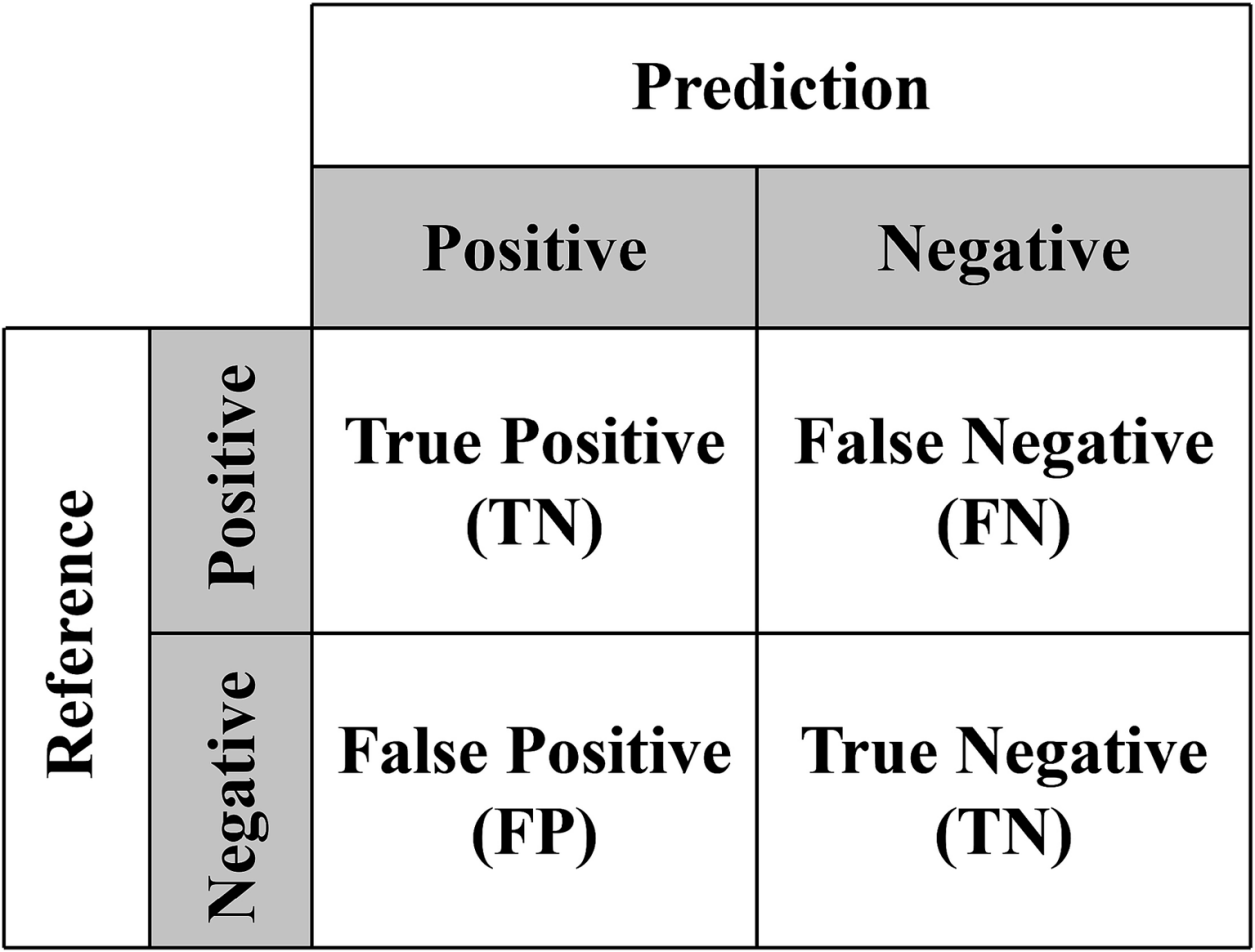
Confusion matrix.

The confusion matrix is a tool used to evaluate the overall performance of a model. Each row corresponds to the true category, while each column represents the category predicted by the model. The number in each cell indicates the number of samples that fall into the corresponding row. In this study, the confusion matrix is based on the following definitions: T (True) for correct predictions, F (False) for incorrect predictions, P (Positive) for positive cases, and N (Negative) for negative cases. This framework yields four key metrics: True Positive (TP), True Negative (TN), False Positive (FP), and False Negative (FN) (see Figure 4). The parameters mentioned above are used to calculate the following performance indicators: accuracy = (TP + TN)/(TP + FP + FN + TN); sensitivity = TP/(TP + FN); precision = TP/(TP + FP); specificity = TN/(TN + FP); recall = TP/TP + FN; NPV = TN/(TN + FN); *F*1-score = (2 * Precision * recall)/(Precision + recall); and AUC.

To further evaluate the generalizability of the classifiers at the individual level and to mitigate the risk of subject-related information leakage inherent in random trial-level splitting, we additionally employed a Leave-One-Subject-Out (LOSO) cross-validation scheme. In this procedure, for each of the 140 participants, all trials belonging to that participant were held out as an independent test set, while data from the remaining 139 participants constituted the training set. This ensures that no trials from the same individual appear in both the training and test sets, thereby providing a more rigorous estimate of the model’s ability to generalize to unseen subjects. To maintain consistency and comparability with the primary analysis, the optimal hyper-parameters identified through the aforementioned 10-fold cross-validation strategy and the optimal feature subsets selected by the Recursive Feature Elimination (RFE) algorithm were adopted. The same six classification models and performance metrics were used to evaluate the LOSO results.

## 3 Results

### 3.1 General statistical analysis

**Figure 5:**
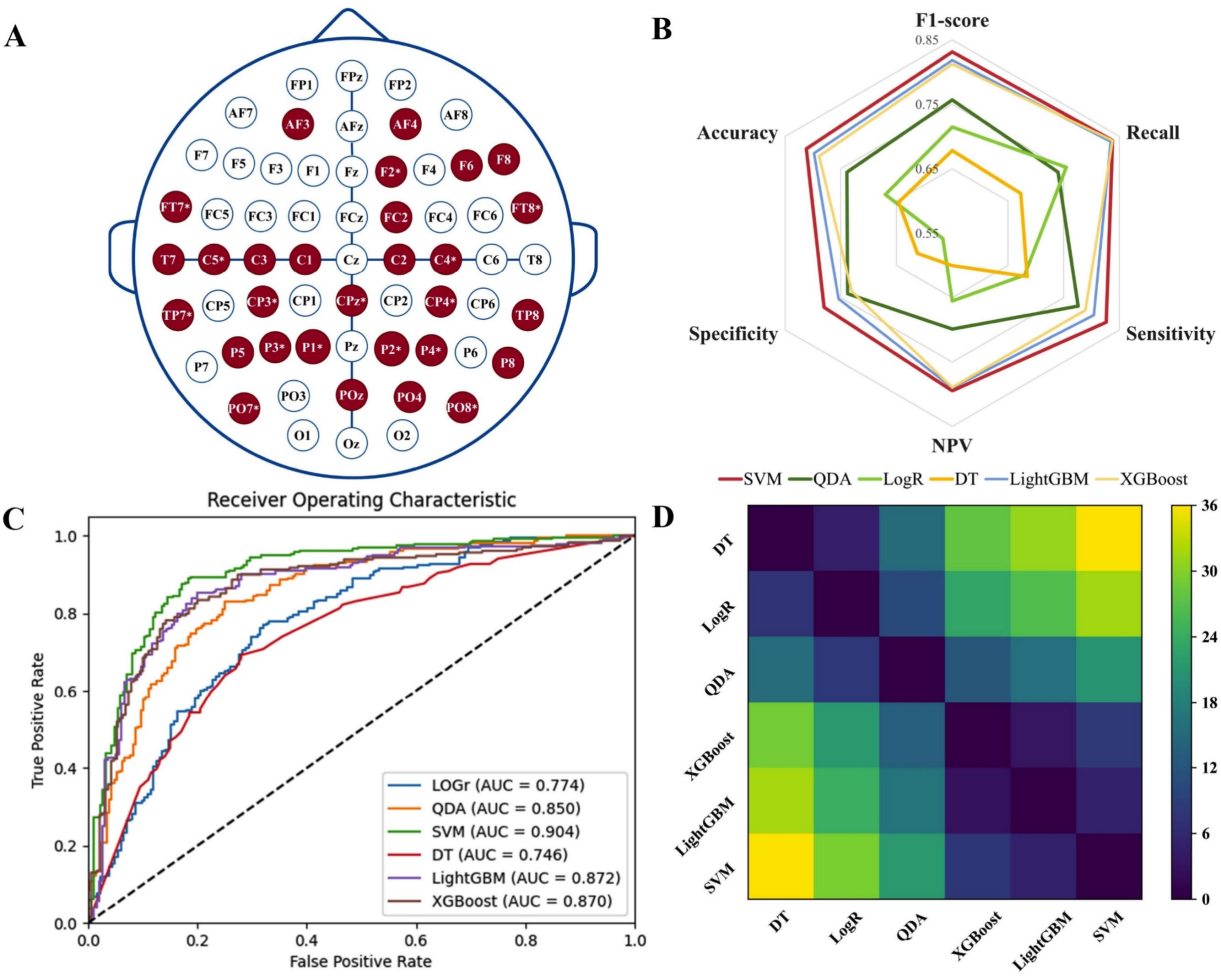
Results of the PSD analysis. **(A)** Red circles illustrate the feature distribution of the PSD. **(B)** Performance metrics of the six classifiers utilizing PSD as the classification feature. **(C)** AUC curves for the six classifiers employing PSD as the classification feature. **(D)** Coefficients of variation for the accuracy and *F*1-score results of the six machine learning models. The upper half presents the Bonferroni follow-up test results for accuracy, while the lower half displays the Bonferroni follow-up test results for *F*1-scores.

**Figure 6:**
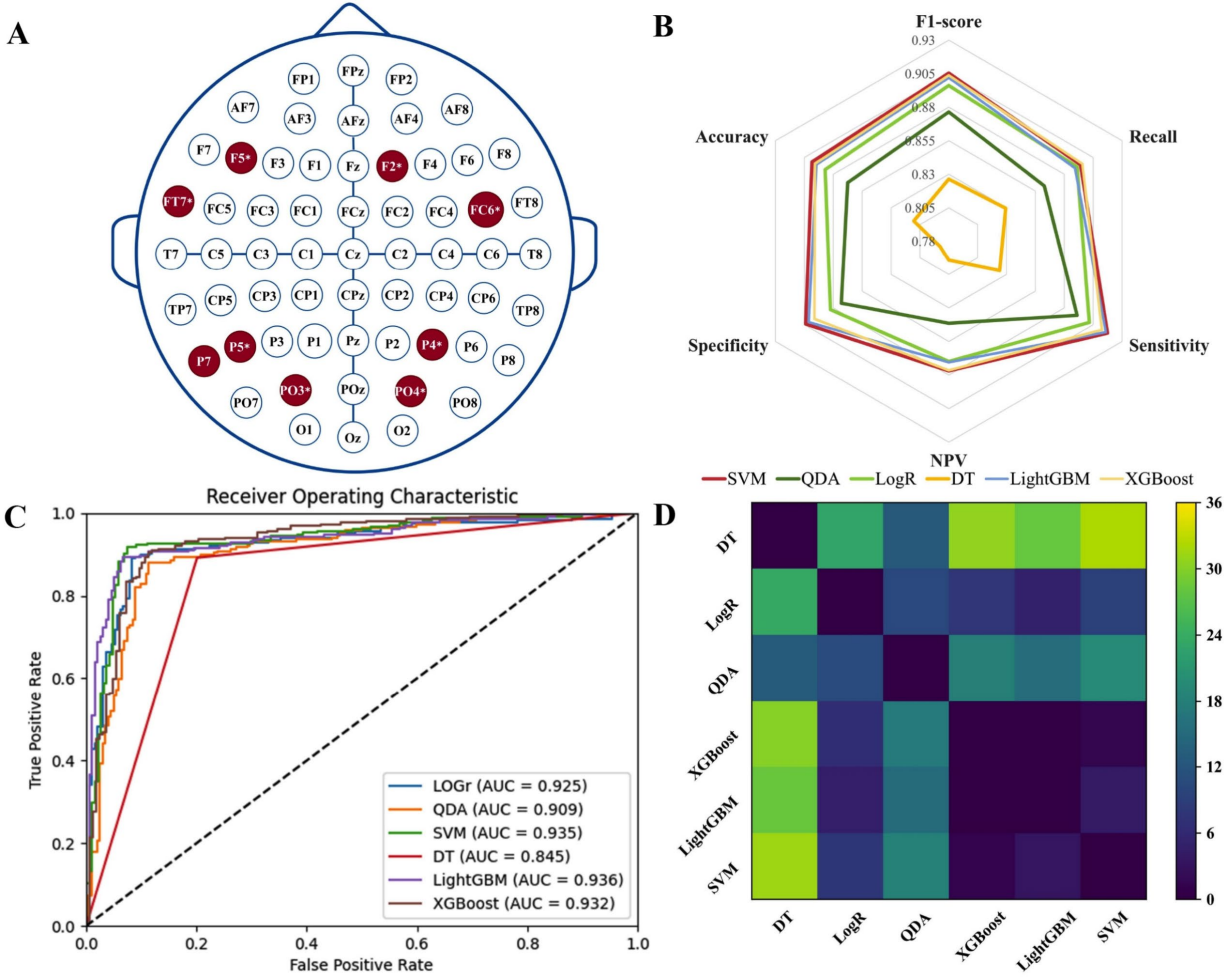
Results of the approximate entropy analysis. **(A)** Red circles illustrate the feature distribution of the approximate entropy. **(B)** Performance metrics of the six classifiers utilizing approximate entropy as the classification feature. **(C)** AUC curves for the six classifiers employing approximate entropy as the classification feature. **(D)** Coefficients of variation for the accuracy and *F*1-score results of the six machine learning models. The upper half presents the Bonferroni follow-up test results for accuracy, while the lower half displays the Bonferroni follow-up test results for *F*1-scores.

**Figure 7:**
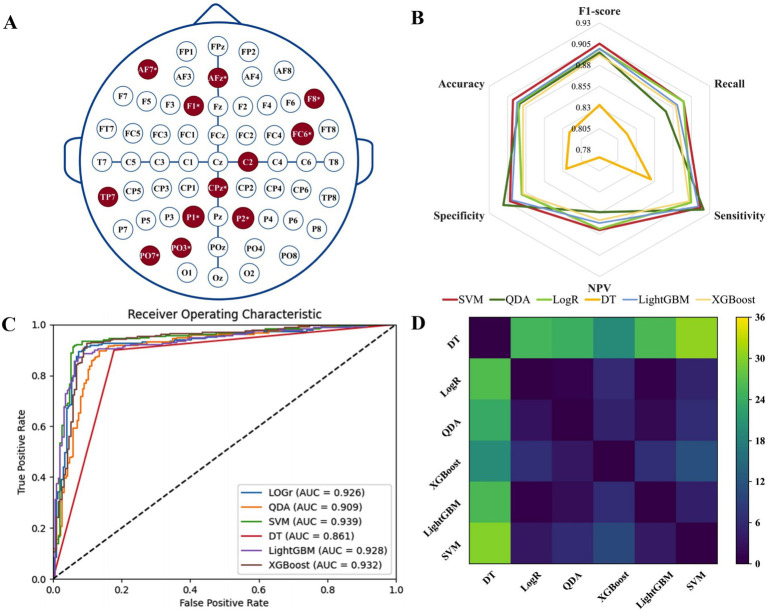
Results of the sample entropy analysis. **(A)** Red circles illustrate the feature distribution of the sample entropy. **(B)** Performance metrics of the six classifiers utilizing sample entropy as the classification feature. **(C)** AUC curves for the six classifiers employing approximate entropy as the classification feature. **(D)** Coefficients of variation for the accuracy and *F*1-score results of the six machine learning models. The upper half presents the Bonferroni follow-up test results for accuracy, while the lower half displays the Bonferroni follow-up test results for *F*1-scores.

Independent samples *t*-tests were conducted to compare the high and low creativity groups across various feature subsets. The analysis revealed significant differences between the high and low creativity groups in terms of PSD at the following electrodes: P3, P4, C4, FT7, C5, TP7, CP3, P1, PO7, PO8, P2, CPz, CP4, FT8, F2. Additionally, significant differences were observed in approximate entropy at the electrodes: P4, FC6, F5, FT7, P5, PO3, PO4, F2. Furthermore, in terms of sample entropy, significant differences were found at the electrodes: FC6, F8, AF7, AFz, F1, P1, PO7, PO3, P2, CPz (see Table 2, Figures 5–7).

**Table 2:** Independent samples *t*-test results for PSD, approximate entropy, and sample entropy for high and low creativity groups.

Electrode	PSD					Approximate entropy			Sample entropy		
	*t*	*p*		*t*	*p*		*t*	*p*		*t*	*p*
C3	−1.414	0.158	P5	1.098	0.272	P7	0.574	0.566	**FC6**	3.42	0.001
T7	−1.552	0.121	**PO7**	−1.988	0.047	**P4**	5.168	<0.001	**F8**	8.368	<0.001
**P3**	2.042	0.041	POz	−0.593	0.553	**FC6**	3.42	0.001	**AF7**	7.429	<0.001
**P4**	6.395	<0.001	PO4	−1.845	0.065	**F5**	5.579	<0.001	**AFz**	6.065	<0.001
P8	−1.044	0.297	**PO8**	−5.779	<0.001	**FT7**	4.927	<0.001	**F1**	8.294	<0.001
**C4**	2.085	0.037	**P2**	3.932	<0.001	**P5**	3.664	<0.001	TP7	−0.007	0.995
FC2	−0.637	0.524	**CPz**	4.070	<0.001	**PO3**	9.612	<0.001	**P1**	6.585	<0.001
F8	−1.642	0.101	**CP4**	2.268	0.023	**PO4**	7.044	<0.001	**PO7**	3.997	<0.001
AF3	−0.767	0.443	TP8	−0.127	0.899	**F2**	2.744	0.006	**PO3**	9.612	<0.001
**FT7**	2.811	0.005	C2	1.867	0.062				**P2**	5.854	<0.001
C1	1.408	0.159	**FT8**	3.127	0.002				**CPz**	5.928	<0.001
**C5**	−2.518	0.012	F6	−1.138	0.255				C2	1.602	0.109
**TP7**	−5.419	<0.001	AF4	0.571	0.568						
**CP3**	−4.203	<0.001	**F2**	−2.322	0.020						
**P1**	2.387	0.017									

Significant values are in [bold]. PSD, power spectral density.

### 3.2 Machine learning classification results: PSD

In comparing the seven metrics of the six machine learning models, it was found that the SVM, LightGBM, and XGBoost models yielded superior recognition results. The *F*1-scores were 83.1, 81.8, and 81.2%, respectively; the accuracies were 81.2, 79.8, and 78.9%; and the sensitivities were 83.8, 83.4, and 83.8% (see Table 3, Figure 5B).

**Table 3:** PSD machine learning classification results (*M* ± SD) %.

Metric	SVM	QDA	LogR	DT	LightGBM	XGBoost
*F*1-score	83.1 ± 1.7	75.7 ± 2.6	71.52.1	67.8 ± 2.4	81.8 ± 2.4	81.2 ± 1.6
Accuracy	81.2 ± 1.7	73.9 ± 2.4	67.0 ± 2.2	64.6 ± 2.3	79.8 ± 2.3	78.9 ± 1.5
Sensitivity	83.8 ± 3.0	74.0 ± 4.1	75.5 ± 3.5	67.3 ± 3.2	83.4 ± 3.3	83.8 ± 2.4
Precision	82.6 ± 2.7	77.6 ± 2.6	68.0 ± 2.3	68.4 ± 2.8	80.4 ± 3.3	78.9 ± 2.5
Specificity	78.0 ± 3.8	73.8 ± 3.6	56.7 ± 3.4	61.2 ± 3.6	75.4 ± 3.9	73.0 ± 3.0
NPV	79.5 ± 3.2	69.9 ± 3.9	65.5 ± 4.6	60.0 ± 3.3	79.0 ± 3.8	78.9 ± 2.9

NPV, negative predictive value; SVM, support vector machine; QDA, quadratic discriminant analysis; LogR, logistic regression; DT, decision tree; XGBoost, extreme gradient boosting; LightGBM, light gradient boosting machine.

The six classifiers outperformed random guessing in both accuracy and *F*1-score. SVM achieved optimal results across seven metrics: *F*1-score, accuracy, sensitivity, precision, specificity, NPV, and AUC, with a sensitivity of 83.8% for classifying highly creative trials. Friedman’s test on the results of the six classifiers revealed significant differences across all seven evaluation indices. The results were as follows: *F*1-score: *χ*^2^ (5) = 2153.484, *p* < 0.001; accuracy: *χ*^2^ (5) = 2192.722, *p* < 0.001; sensitivity: *χ*^2^ (5) = 2002.243, *p* < 0.001; precision: *χ*^2^ (5) = 1959.392, *p* < 0.001; specificity: *χ*^2^ (5) = 1905.773, *p* < 0.001; NPV: *χ*^2^ (5) = 2051.743, *p* < 0.001; AUC value: *χ*^2^ (5) = 2223.368, *p* < 0.001. A Bonferroni follow-up test indicated that the classifiers ranked in the following order based on *F*1-scores, accuracy, and AUC values: SVM > LightGBM > XGBoost > DQA > LogR > DT (see Figure 5).

### 3.3 Machine learning classification results: approximate entropy

In comparing the seven metrics across six machine learning models, it was observed that the recognition results for the SVM, LightGBM, and XGBoost models were superior. The *F*1-scores were 90.5, 90.2, and 90.4%, respectively; the accuracies were 89.8, 89.5, and 89.6%; and the sensitivities were 89.4, 88.9, and 89.6% (see Table 4, Figure 6B).

**Table 4:** Approximate entropy machine learning classification results (*M* ± SD) %.

Metric	SVM	QDA	LogR	DT	LightGBM	XGBoost
*F*1-score	90.5 ± 1.3	87.6 ± 1.6	89.6 ± 1.3	82.6 ± 1.5	90.2 ± 2.1	90.4 ± 1.0
Accuracy	89.8 ± 1.3	86.8 ± 1.7	88.7 ± 1.4	81.0 ± 1.5	89.5 ± 2.1	89.6 ± 1.1
Sensitivity	89.4 ± 2.0	86.3 ± 2.2	89.1 ± 1.7	82.9 ± 2.1	88.9 ± 3.0	89.6 ± 1.8
Precision	91.7 ± 1.7	89.1 ± 2.0	90.2 ± 2.1	82.4 ± 2.0	91.6 ± 2.6	91.3 ± 1.5
Specificity	86.5 ± 4.2	88.5 ± 4.2	80.9 ± 3.9	89.3 ± 4.5	89.4 ± 4.2	89.6 ± 1.7
NPV	87.7 ± 2.4	84.1 ± 2.3	87.1 ± 2.1	79.4 ± 2.2	87.1 ± 3.5	87.7 ± 2.1

The six classifiers outperformed random guessing in both *F*1-score and accuracy. SVM achieved optimal results across six metrics: *F*1-score, accuracy, sensitivity, precision, specificity, NPV, and AUC. Notably, the sensitivity for classifying highly creative trials reached 89.4%. Friedman’s test on the performance of the six classifiers revealed significant differences across all seven evaluation indices. The results were as follows: *F*1-score: *χ*^2^ (5) = 1513.274, *p* < 0.001; accuracy: *χ*^2^ (5) = 1560.164, *p* < 0.001; sensitivity: *χ*^2^ (5) = 1211.787, *p* < 0.001; precision: *χ*^2^ (5) = 1334.464, *p* < 0.001; specificity: *χ*^2^ (5) = 1337.109, *p* < 0.001; NPV: *χ*^2^ (5) = 1339.362, *p* < 0.001; AUC value: *χ*^2^ (5) = 1394.150, *p* < 0.001. A Bonferroni follow-up test indicated that the classifiers ranked in the following order based on *F*1-scores, accuracy, and AUC values: SVM > XGBoost > LightGBM > LogR > DQA > DT (see Figure 6).

### 3.4 Machine learning classification results: sample entropy

The comparison of seven metrics across six machine learning models indicates that the SVM, LightGBM, QDA, LogR, and XGBoost models yield superior recognition results. The *F*1-scores were 90.5, 90.0, 89.5, 89.9, and 89.2%, respectively; the accuracies were 89.8, 89.2, 88.9, 89.0, and 88.4%; and the sensitivities were 89.4, 88.6, 87.0, 89.4, and 88.2% (see Table 5, Figure 7B).

**Table 5:** Sample entropy machine learning classification results (*M* ± SD) %.

Metric	SVM	QDA	LogR	DT	LightGBM	XGBoost
*F*1-score	90.5 ± 1.3	89.5 ± 1.6	89.9 ± 1.3	83.3 ± 1.8	90.0 ± 2.0	89.2 ± 1.3
Accuracy	89.8 ± 1.4	88.9 ± 1.5	89.0 ± 1.3	82.1 ± 1.7	89.2 ± 2.1	88.4 ± 1.3
Sensitivity	89.4 ± 1.8	87.0 ± 2.2	89.4 ± 1.8	81.7 ± 2.7	88.6 ± 2.8	88.2 ± 2.0
Precision	91.7 ± 1.8	92.2 ± 1.6	90.5 ± 1.5	85.0 ± 2.4	91.4 ± 2.5	90.2 ± 1.8
Specificity	90.2 ± 2.2	91.1 ± 1.8	88.6 ± 1.9	82.5 ± 2.9	89.9 ± 3.1	88.4 ± 2.2
NPV	87.5 ± 2.1	85.4 ± 2.3	87.4 ± 2.2	78.9 ± 2.8	86.7 ± 3.1	86.3 ± 2.2

The six classifiers outperformed random guessing in both *F*1-score and accuracy. SVM achieved optimal results across six metrics: *F*1-score, accuracy, sensitivity, precision, specificity, NPV, and AUC. Notably, the sensitivity for classifying highly creative trials reached 89.4%. Friedman’s test on the results of the six classifiers indicated significant differences among them across all seven evaluation metrics. The results were as follows: *F*1-score: *χ*^2^ (5) = 1166.958, *p* < 0.001; accuracy: *χ*^2^ (5) = 1187.524, *p* < 0.001; sensitivity: *χ*^2^ (5) = 1192.04, *p* < 0.001; precision: *χ*^2^ (5) = 1216.769, *p* < 0.001; specificity: *χ*^2^ (5) = 1237.493, *p* < 0.001; NPV: *χ*^2^ (5) = 1099.15, *p* < 0.001; AUC: *χ*^2^ (5) = 1455.151, *p* < 0.001 (see Figure 7).

### 3.5 Leave-one-subject-out validation

To address the potential issue of subject-related information leakage inherent in random trial-level splitting, we conducted an additional validation using a Leave-One-Subject-Out (LOSO) cross-validation scheme. In this procedure, data from each of the 140 participants were sequentially held out as an independent test set, while the remaining 139 participants constituted the training set. This ensures that no trials from the same individual appear in both the training and test sets, thereby providing a more rigorous and generalizable estimate of classifier performance at the individual level.

To maintain computational feasibility, the optimal hyper-parameters determined via the 10-fold cross-validation strategy were adopted for each classifier. Similarly, the optimal feature subsets identified by the Recursive Feature Elimination (RFE) algorithm were employed. Six classifiers (SVM, QDA, LogR, DT, XGBoost, and LightGBM) were evaluated across the three feature sets (alpha PSD, approximate entropy, and sample entropy). Classification performance was assessed using the same metrics reported in the preceding sections.

As anticipated, the LOSO validation yielded lower performance metrics compared to the trial-level random split (see Table 6), reflecting the increased difficulty of generalizing across unseen individuals rather than across trials from familiar subjects. Nevertheless, the SVM classifier continued to demonstrate the most robust performance. Specifically, when utilizing approximate entropy as the classification feature, the SVM achieved an *F*1-score of 82.4% and an accuracy of 81.5%. Similarly, with sample entropy, the SVM attained an *F*1-score of 82.1% and an accuracy of 81.2%. These results remain substantially above chance level (50%) and confirm that the classifier captures creativity-related neural signatures rather than subject-specific idiosyncrasies.

**Table 6:** Leave-one-subject-out validation results (*M* ± SD) % for six machine learning classifiers across three feature sets.

Feature	Metric	SVM	QDA	LogR	DT	LightGBM	XGBoost
Alpha PSD	*F*1-score	74.2 ± 2.1	68.5 ± 2.8	65.3 ± 2.4	61.2 ± 2.6	73.1 ± 2.2	72.5 ± 1.9
	Accuracy	73.5 ± 2.0	67.1 ± 2.6	63.8 ± 2.3	59.4 ± 2.5	72.0 ± 2.1	71.4 ± 1.8
	Sensitivity	75.8 ± 3.2	66.4 ± 4.0	68.2 ± 3.6	60.1 ± 3.4	74.5 ± 3.1	75.2 ± 2.8
	Precision	72.8 ± 2.8	70.8 ± 2.9	62.9 ± 2.5	62.3 ± 2.9	71.8 ± 3.0	70.1 ± 2.6
	Specificity	71.2 ± 3.6	67.8 ± 3.5	59.4 ± 3.3	58.7 ± 3.5	69.5 ± 3.4	67.6 ± 3.1
	AUC	0.812	0.758	0.721	0.698	0.801	0.795
Approximate entropy	*F*1-score	**82.4 ± 1.8**	78.6 ± 2.1	80.1 ± 1.9	74.2 ± 2.3	81.8 ± 2.0	81.5 ± 1.7
	Accuracy	**81.5 ± 1.7**	77.2 ± 2.0	79.3 ± 1.8	72.8 ± 2.2	80.9 ± 1.9	80.6 ± 1.6
	Sensitivity	81.8 ± 2.5	76.5 ± 2.8	79.4 ± 2.4	73.5 ± 2.7	80.5 ± 2.6	81.2 ± 2.3
	Precision	83.1 ± 2.2	80.8 ± 2.4	81.0 ± 2.3	75.0 ± 2.5	83.2 ± 2.4	82.0 ± 2.1
	Specificity	81.2 ± 2.8	78.0 ± 2.9	79.2 ± 2.6	72.1 ± 3.0	80.3 ± 2.7	80.0 ± 2.4
	AUC	**0.874**	0.838	0.851	0.805	0.868	0.865
Sample entropy	*F*1-score	**82.1 ± 1.9**	80.5 ± 2.0	81.2 ± 1.8	75.0 ± 2.4	81.5 ± 2.1	80.8 ± 1.8
	Accuracy	**81.2 ± 1.8**	79.5 ± 1.9	80.1 ± 1.7	73.5 ± 2.3	80.6 ± 2.0	79.9 ± 1.7
	Sensitivity	81.5 ± 2.4	78.8 ± 2.6	80.5 ± 2.3	72.8 ± 2.8	80.2 ± 2.5	79.8 ± 2.4
	Precision	82.8 ± 2.3	82.4 ± 2.2	82.0 ± 2.1	77.4 ± 2.6	82.9 ± 2.3	81.8 ± 2.2
	Specificity	80.9 ± 2.7	80.2 ± 2.6	79.7 ± 2.5	74.2 ± 2.9	81.0 ± 2.6	80.0 ± 2.5
	AUC	**0.871**	0.855	0.862	0.812	0.865	0.858

Values in bold indicate the best performance within each feature set.

Notably, the entropy-based features (approximate entropy and sample entropy) again outperformed alpha PSD under the LOSO protocol, reinforcing the conclusion that nonlinear complexity measures provide more generalizable and noise-resistant markers of creative thinking. Although the absolute performance decreased relative to the trial-level analysis, the rank order of classifiers remained largely consistent: SVM, XGBoost, and LightGBM continued to outperform QDA, LogR, and DT.

These findings suggest that while the trial-level cross-validation results reported in Sections 3.2–3.4 reflect the upper bound of classification performance within the dataset, the LOSO results provide a conservative yet realistic estimate of the model’s capacity to classify creativity levels in novel individuals. Future studies aiming to develop clinically or educationally applicable creativity assessment tools should prioritize subject-independent validation schemes such as LOSO.

## 4 Discussion

The purpose of this study is to investigate whether machine learning techniques can effectively classify levels of creativity by comparing various machine learning methods. Utilizing EEG technology and principles of signal theory, this research employs alpha band PSD, approximate entropy, and sample entropy extracted from EEG data as classification features. The originality ratings (high and low) of the multipurpose task items serve as classification labels. To facilitate a comprehensive comparison of the classification performance of different machine learning models on creative thinking EEG data, six models were selected for this study: SVM, QDA, LogR, DT, XGBoost, and LightGBM. Notably, a balanced accuracy of 90.5% was achieved when SVM was used as the classifier with either approximate entropy or sample entropy as the classification feature. This study confirms the effectiveness of machine learning classification techniques in applications involving EEG signals related to creative thinking.

Recently, numerous studies have used machine learning, deep learning, and artificial intelligence techniques for the classification of EEG data, achieving commendable classification performance (He et al., 2025; Cao et al., 2025; Kirik, 2024). However, there are relatively few studies that apply these methods to the field of creativity. Sasaki et al. utilized machine learning techniques to investigate musical creativity, analyzing EEG activity to distinguish between the performance demands faced by guitarists—improvisation vs. scale—resulting in a classification accuracy of 75% (Sasaki et al., 2019). Stevens et al. collected EEG data from participants as they completed an AUT, and applied QDA and SVM techniques to categorize the participants’ creativity levels, achieving a classification accuracy of 82.3% (Stevens and Zabelina, 2020). In comparison to previous studies, the method proposed in this research demonstrates superior classification performance.

The two information entropies classify data more effectively than the alpha band power spectral density. This may be attributed to the fact that information entropy exhibits superior noise immunity as a measure of signal complexity (Richman et al., 2004). Furthermore, information entropy can more comprehensively leverage the information within the data, characterize the signal, and provide a measure of the overall information content of the EEG signal. In comparison to power spectral density, information entropy offers several advantages in extracting the informational characteristics of EEG data, particularly in the presence of noise. Ueno et al. (2015) found that the information entropy of EEG signals significantly differed between high and low creativity groups. A previous study utilizing resting-state functional MRI technology similarly confirmed the correlation between creative thinking and brain information entropy (Shi et al., 2020). Information entropy may serve as a key variable of interest in future research related to creative thinking (Kaur et al., 2021). However, approximate entropy and sample entropy describe the entropy of the stimulus window as a whole, focusing solely on the complexity of the entire time series. Multiscale entropy can more effectively capture the characteristics of signal changes across different time scales by calculating entropy values at various scales (Kaur et al., 2020; Wu et al., 2014). In future research, multiscale entropy or other forms of information entropy could be incorporated into model training to categorize creative thinking from a temporal perspective. Reason: Improved vocabulary, clarity, and technical accuracy while correcting grammatical and punctuation errors.

Compared to the low creativity group, the high creativity group exhibited significant differences in parietal power spectral density. This indicates a higher activation of the parietal lobe during creative activities in the high creativity group, consistent with the findings of previous studies (Boccia et al., 2015). These results suggest that the parietal cortex plays a more critical role in creative processes. Prior research has indicated that the parietal cortex is involved in cognitive functions such as long-term memory, cognitive control, and association (Boccia et al., 2015; Dietrich, 2004; Olivetti Belardinelli et al., 2009), all of which are essential for generating creative thought. Furthermore, the item versatility paradigm employed in the present study requires participants to envision as many novel uses for conventional items as possible. This may facilitate a process of retrieving information related to these items from long-term memory while suppressing associations with their common uses during the ideation phase. Consequently, the high creativity group in this study may have engaged more neural resources in the parietal cortex during creative tasks, which supported their ability to produce superior creative responses.

Both information entropies were significantly higher in the frontal and parietal lobes of the high creativity group compared to the low creativity group. This finding aligns with previous research and further supports the notion that elevated creativity levels are associated with increased neural network activation and flexibility in the cerebral cortex (Ueno et al., 2015). The brain regions identified as having high information entropy in this study are typically regarded as being linked to creative thinking (Benedek, 2018). These regions play crucial roles in various cognitive tasks; for instance, both the prefrontal cortex and parietal cortex are essential for long-term memory and cognitive control processes (Boccia et al., 2015; Fink et al., 2007). It has been suggested that the human brain, as a dynamic functional system, exhibits sustained fluctuations in brain activity, and that the extent of these fluctuations is related to the brain’s information-processing capacity (Xue et al., 2019). A broader range of fluctuations may facilitate the understanding and prediction of variable external events (Shi et al., 2020). Consequently, higher information entropy indicates more irregular brain activity and enhanced information processing capabilities. Furthermore, this finding may suggest that the brains of individuals in the highly creative group are more adaptable to external stimuli, enabling them to process complex information and changes more effectively, thereby demonstrating greater creativity and improved problem-solving abilities in creative thinking tasks. Reason: Improved clarity, vocabulary, and technical accuracy while maintaining the original meaning.

Among the six models, SVM demonstrated the best classification performance, while DT exhibited the poorest results. This disparity may be attributed to several factors: (1) The complexity of EEG data: The feature space of EEG data is high-dimensional, and SVM excels in managing such high-dimensional data. (2) Nonlinearity of EEG data: SVM effectively addresses the nonlinearity of data by mapping input features to a high-dimensional space using kernel functions. In contrast, DT requires more intricate partitioning to tackle nonlinear problems, which can hinder its ability to achieve optimal performance. (3) Robustness: SVM is more resilient to noise interference in EEG signals and demonstrates greater robustness compared to decision trees. While SVM is generally more suitable for analyzing EEG data, this does not imply that DT’s classification performance is always inferior to that of SVM; each algorithm has its own applicable scenarios and limitations. In a classification study involving SVM, DT, and artificial neural networks, DT’s classification accuracy matched that of SVM, despite DT requiring significantly less computation time (Ozden et al., 2015). Furthermore, in another study examining machine learning techniques for depression diagnosis, DT outperformed SVM in terms of depression detection accuracy (Zhao and Feng, 2020). This indicates that DT may be more advantageous than SVM in classification tasks that involve “if, then” logic (Sundarkumar and Ravi, 2015). Therefore, researchers should select and evaluate algorithms based on task requirements, data characteristics, and the algorithms themselves. The findings of this study also serve as a reference for future research regarding the application of machine learning techniques to EEG data and creative thinking domains, as well as guidance on which classification models to choose.

This study investigates the classification performance of various machine learning techniques in assessing levels of creativity, offering a new perspective for the in-depth exploration of creative thinking. However, several issues and limitations persist in this research. First, the study is constrained by the amount of data available and opts to classify based on the number of trials. This approach allows for a larger dataset and enhances model classification performance, thereby mitigating overfitting issues. Nonetheless, there is a notable absence of classification models that consider individual perspectives. Future research should aim to recruit more participants and gather additional data from each individual to facilitate creative identification and classification through deep learning models from a personal standpoint. Second, while the present study was designed to evaluate the independent discriminative contribution of each feature type—an approach that preserves theoretical interpretability—future research would benefit from exploring feature fusion strategies. Specifically, combining spectral (PSD) and non-linear (ApEn, SampEn) features into a unified classification framework could provide complementary information about the neural dynamics of creative cognition. Several multi-modal fusion approaches merit investigation (Huang et al., 2026): (1) Early fusion (feature concatenation): Directly merging all feature vectors before classification, which may capture joint distributions that single-feature models miss; (2) Late fusion (ensemble learning): Training separate classifiers on each feature type and aggregating predictions through majority voting or stacking, preserving feature-specific sensitivity; (3) Attention-based fusion in deep learning models: Allowing the model to learn which features from which channels and frequency bands are most relevant at each classification stage. Third, this study utilized EEG data as a measurement tool. However, EEG technology suffers from poor spatial resolution, which limits its ability to accurately characterize the activation of brain regions involved in creative activities. Future research could benefit from using functional magnetic resonance imaging (fMRI) data as classification features for machine learning, allowing for a more precise identification and classification of localized brain regions. Fourth, the present study employed the CAT to evaluate the originality of creative responses (Amabile, 1982). While CAT is widely regarded as the gold standard for creativity assessment, it is an inherently subjective approach that relies on human judgment. Despite the satisfactory inter-rater reliability (ICC = 0.863) observed in this study, the subjectivity inherent in human ratings may introduce variability that cannot be fully eliminated. Moreover, raters’ evaluations may be influenced by their personal understanding of creativity, cultural background, and domain-specific expertise. Future research could complement CAT-based scoring with more objective, computational measures of creativity—such as semantic distance algorithms or machine learning-based automated scoring—to triangulate creativity assessments and reduce reliance on subjective human judgment.

## 5 Conclusion

This study demonstrates the effectiveness of ML classification techniques in analyzing creative EEG signals by comparing multiple evaluation metrics across six ML models: SVM, QDA, LogR, DT, XGBoost, and LightGBM. The classification task is evaluated based on power spectral density, approximate entropy, and sample entropy of EEG signals associated with creative thinking. The results indicate that PSD, approximate entropy, and sample entropy are all effective features for distinguishing between high and low creativity levels, with a particular emphasis on the significance of approximate entropy and sample entropy in this context. Among the six models, SVM, XGBoost, and LightGBM demonstrate superior performance in identifying high and low creativity groups. Future research should focus on exploring individual classification models and incorporating data from various modalities as classification features to enhance classification performance.

## Data Availability

The datasets presented in this study can be found in online repositories. The names of the repository/repositories and accession number(s) can be found below: doi: 10.57760/sciencedb.psych.00751.
